# SARS-CoV-2 main protease targeting potent fluorescent inhibitors: Repurposing thioxanthones

**DOI:** 10.55730/1300-0527.3541

**Published:** 2023-01-05

**Authors:** Gönül S. BATIBAY, Eyüp METİN

**Affiliations:** 1 Department of Chemistry, Faculty of Arts and Sciences, Yıldız Technical University, İstanbul, Turkiye; 2 Department of Materials Science and Technology, Faculty of Science, Turkish-German University, İstanbul, Turkiye

**Keywords:** SARS-CoV-2, COVID-19, drugs, docking, molecular dynamics, repurposing

## Abstract

The coronavirus disease, COVID-19, is the major focus of the whole world due to insufficient treatment options. It has spread all around the world and is responsible for the death of numerous human beings. The future consequences for the disease survivors are still unknown. Hence, all contributions to understand the disease and effectively inhibit the effects of the disease have great importance. In this study, different thioxanthone based molecules, which are known to be fluorescent compounds, were selectively chosen to study if they can inhibit the main protease of SARS-CoV-2 using various computational tools. All candidate ligands were optimized, molecular docking and adsorption, distribution, metabolism, excretion, and toxicity (ADMET) studies were conducted and subsequently, some were subjected to 100 ns molecular dynamics simulations in conjunction with the known antiviral drugs, favipiravir, and hydroxychloroquine. It was found that different functional groups containing thioxanthone based molecules are capable of different intermolecular interactions. Even though most of the studied ligands showed stable interactions with the main protease, para-oxygen-di-acetic acid functional group containing thioxanthone was found to be a more effective inhibitor due to the higher number of intermolecular interactions and higher stability during the simulations.

## 1. Introduction

The coronavirus disease, COVID-19, is defined as an acute respiratory tract disease caused by the severe acute respiratory syndrome coronavirus 2, SARS-CoV-2. The disease was first reported in December 2019 in Wuhan, China. Later the disease was declared as a pandemic by World Health Organization (WHO) in March 2020. Since the first case, the disease has spread all around the world. Globally, as of April 1, 2022, there have been 486,761,597 confirmed cases of COVID-19, including 6,142,735 deaths, reported to WHO^1^. The common symptoms of this disease are reported as fever, fatigue, loss of smell, cough, and in severe cases shortness of breath [1–3]. Currently, there is no specific antiviral drug against SARS-CoV-2. Several vaccines have been developed by various pharmaceutical companies and countries. Even though the vaccines were administered to people around the globe, still many people, like those who live in 3^rd^ world countries, have difficulties to access to these vaccines. In addition, several different mutations were reported. Despite the efforts of humankind, this disease seems to be staying among us for a while. For this reason, SARS-CoV-2 related research still requires attention both today and in the future. In today’s perspective preventing disease transmission and reducing the number of infected people and deaths are high-priority goals. However, controlling the disease in the future requires new approaches which include new detection, sampling, and analytical methods.

It is known that the viruses capable of causing sickness in humans encode proteases. They are responsible for the cleavage of viral polyproteins and hence continuing the viral replication processes. Therefore, proteases are important and ideal targets for viral diseases as they have significant importance in the life cycle of the viruses [3–5].

The crystal structure of SARS-CoV-2 main protease (M^pro^), also known as 3-chymotrypsin like protease (3CL^pro^), with an inhibitor N3 was made publicly available by Jin et al. [6].

N3 is an inhibitor designed with computer-aided drug design (CADD) strategies and can specifically inhibit M^pro^ of several coronaviruses, such as previously encountered SARS-CoV and Middle East Respiratory Syndrome Coronavirus (MERS-CoV) [6–9].

SARS-CoV-2 replicase encodes two polyproteins, namely pp1a and pp1ab, which are required for the production of functional polypeptides that have significant importance in the viral replication and transcription processes. These functional polypeptides are released from the polyproteins as a result of extensive proteolytic processes by M^pro^ [6], [10–12].

**Figure 1:**
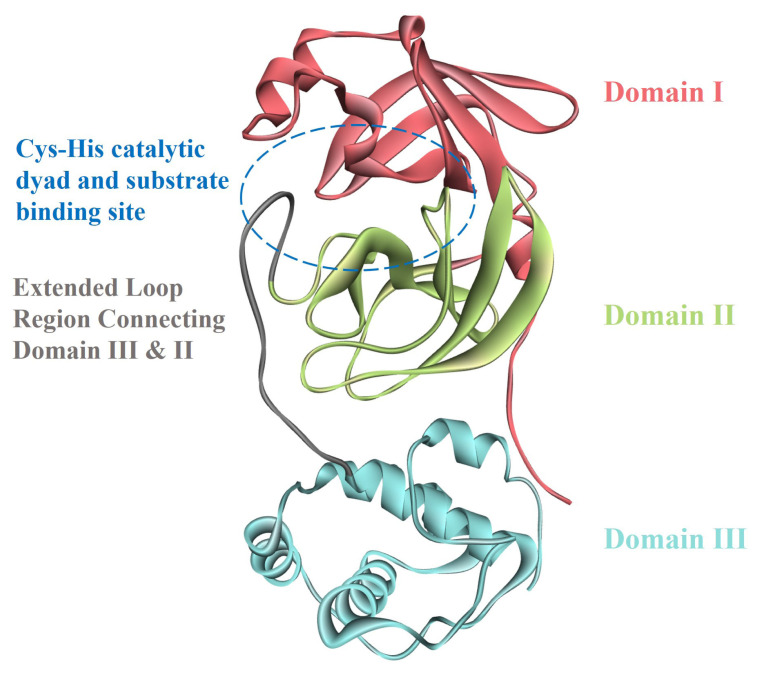
Structure of M^pro^ representing domain I, II, II, extended loop region, and substrate binding site.

M^pro^ is a dimeric protein and contains two symmetric protomer units. Each M^pro^ protomer is composed of three domains. These domains are domain I (D1) (residues 8–101), domain II (D2) (residues 102–184), and domain III (D3) (residues 201–303). D1 and D2 possess an antiparallel β-barrel structure. D3 contains five α-helices, and it is linked to D2 with an extended loop region (residues 185–200). M^pro^ of SARS-CoV-2 contains a Cys-His catalytic dyad and substrate-binding site is positioned in a cavity between D1 and D2 (Figure 1). These descriptive specifications are similar to the M^pro^ of previously encountered coronaviruses [6], [8–10].

Thioxanthone is an organic compound with a heterocyclic structure and can be also classified as the sulfur analog of xanthone. Thioxanthone and its derivatives are versatile compounds and have many applications in different areas due to their exceptional photochemical and photophysical properties [13–14]. Initially, they were utilized as photoinitiators in free radical polymerization [15–19]. Later, the science benefited from the exceptional photochemical and photophysical properties of these molecules in various biological and biochemical systems such as, in DNA binding studies, in cytotoxicity studies, in antibacterial systems, and as drug analogues [20–23]. In past years, several different analogues of thioxanthone based molecules were synthesized with the aim of antitumor efficiency and some other biological activities such as antiallergic, antibiotics, and monoamine oxidase (MAO) inhibitors [13].

In silico studies are successful methods to target biological compounds and have been used in various studies throughout the years. Predominantly used in silico approaches include molecular docking and molecular dynamics simulations. There have been numerous studies for CADD of SARS-CoV-2 and these studies mainly focused on the known drugs and/or blind searching the chemical databases as it was an urgent issue at beginning of the pandemic.

Several other targeted and planned approaches based on the knowledge in the literature were also employed to understand the inhibitory effect of various compounds against SARS-CoV-2. It was proposed previously by Gerçek et. al [24] that some of the SARS-CoV-2 targeting molecules bear three different constituents on the main core such as substituted aromatic rings, *p*-florothiophene and a nitrogen. Coumarin derivatives are also a good example in the literature among the tested molecules against SARS-CoV-2 as they are known to be versatile organic compounds [25–26].

In this study, seven different molecules with thioxanthone core were chosen selectively based on the investigated photophysical and photochemical properties in literature [27–29]. As these molecules possess fluorescent properties [27–29], it is believed that this field of study could benefit from their exceptional photochemical properties. Besides the fluorescent properties of thioxanthone derivatives, these species contain functional groups which are capable of making intermolecular interactions such as hydrogen bonding, π-π interactions, π-alkyl interactions, and more. As reported in the literature, due to the ease of synthesis, thioxanthone based molecules can be also functionalized with different functional groups with desired properties. We used density functional theory (DFT) to optimize thioxanthone based ligands and these structures were used in the molecular docking of M^pro^. After molecular docking the best-performing molecules were determined. Best docking poses of determined molecules were retrieved, and 100 ns molecular dynamics simulations were conducted. Additionally, ADMET properties of thioxanthone derivatives were studied. In conjunction with the thioxanthone derivatives, two known antiviral drugs, favipiravir (FAV) and Hydroxychloroquine (HCQ) were also used in our study as they were administered to the people during the initial stages of the pandemic [30]. Stability of keto and enol forms of favipiravir in water environment was compared and most stable conformer was determined. Most stable conformer of FAV and HCQ were also subjected to molecular docking and subsequent MD simulations to compare their performance with thioxanthone derivatives. At the end of the conducted studies, promising results were obtained and presented to the literature.

## 2. Methodology

### 2.1. Density functional theory (DFT) calculations

Ligands were optimized with Gaussian 16 Revision B.01 software package by density functional theory (DFT). Calculations were carried out at B3LYP [31–33] level and 6–31+G(d,p) basis set was used for all atoms. As reported earlier by Metin et al. [34] adding a larger basis set for the S atom in the DFT calculations of thioxanthone based molecules leads to a good agreement with the experimental results. For this reason, a larger basis set, 6–311++G(3df,3pd), was added for S, F, and Cl atoms. Initially, preoptimization calculations were carried out with the initially created structures. Then, possible rotations around the single bonds were taken into account and these structures were reoptimized. Optimized structures were frequency checked and no imaginary frequency was noted. The minimum energy conformer of each ligand was selected for docking studies. To mimic the biological environment, all optimization calculations were carried out in the water (ɛ = 78.3553) by using the integral equation formalism polarizable continuum model (IEF-PCM) as implemented in Gaussian 16.

### 2.2. Docking

Docking studies were carried out with AutoDock Vina 1.1.2 [35]. As the docking studies require both protein and ligand preparation before running calculations; we used AutoDockTools 1.5.7 (ADT), which is distributed along with the MGLTools 1.5.7 (https://ccsb.scripps.edu/mgltools/), to prepare the protein and the ligands.

BIOVIA Discovery Studio Visualizer 2020 software was used for visualization and investigation purposes throughout the study.

Initially, the crystal structure of SARS-CoV-2 main protease (M^pro^) in complex with N3 [6] (PDB ID: 6LU7) was downloaded from Protein Data Bank in .pdb format. The crystal structure of the protein contains an inhibitor called N3, water molecules and it has missing hydrogens. The .pdb file was opened with ADT and the protein preparation was established by; (i) removing the attached ligand, (ii) removing the surrounding water molecules, (iii) adding missing polar hydrogens, and then (iv) Gasteiger charges. The prepared structure was converted to .pdbqt format to be used in AutoDock Vina calculations.

The position of the active site was determined by the central coordinates of N3 inhibitor in the crystal structure as x: −10.711837, y: 12.411388, z: 68.831286. A cubic grid box, that covers the whole active site, was created by adjusting the spacing as 1 Å and setting the number of points in x,y, and z dimensions as 22 × 22 × 22, respectively. A configuration file was created to run AutoDock Vina with the following parameters: exhaustiveness as 24, the number of modes as 20, and energy range as 2 kcal mol^−1^.

### 2.3. ADMET, drug likeness and medicinal chemistry friendliness

ADME is an abbreviation in pharmacokinetics and pharmacology for “absorption, distribution, metabolism, and excretion”, and it is about the disposition of a pharmaceutical compound within an organism. These all criteria influence the drug levels and kinetics of drug exposure to the living tissues and hence influence the performance and pharmacological activity of the compounds as a drug. Often toxicological properties of a drug are considered to have a vital role in drug design strategies. When toxicity studies accompany ADME studies, the abbreviation is transformed into ADMET where T stands for toxicology.

All main protease-targeting ligands were checked for their absorption, distribution, metabolism, excretion, and toxicity (ADMET) properties, drug-likeness, and medicinal chemistry friendliness with SwissADME [36–38] and pkCSM [39] tools.

### 2.4. Molecular dynamics (MD) simulations

GROMACS 2020.1 software package was used for molecular dynamics simulations. Initially, all molecules including protein and ligands were prepared for the molecular dynamics simulations. At the initial state of the simulation, topology for the protein and the ligands were generated. Subsequently, the protein-ligand complexes were built. A box around the complexes was created and the simulation system was solvated. The created system was energy minimized and equilibrated. Lastly, successful MD production runs were conducted.

During MD runs CHARMM36 all-atom force field and TIP3P water model were used. We benefited from CGenFF server for the ligand preparations [40–44]. The detailed parameters of the created systems are as followed. The entire protein-ligand complex was centered in a dodecahedron box at least 1 nm from the edge of the box. Then the system is solvated. Na^+^ and Cl^−^ ions were added to neutralize the system and set the ion concentration to 0.15 M to obtain a physiological environment. Following the preparation of the system, first, the system was energy minimized. Then the minimized structure was equilibrated by NVT and NPT ensembles for 200 ps, respectively. Verlet cutoff scheme and Particle Mesh Ewald for long-range electrostatics (PME) were used with 1.2 nm cutoff. Modified Berendsen thermostat (V-rescale) was used and the temperature was set to 310 K. For the NPT, pressure coupling with Berendsen was conducted. After successful NVT and NPT equilibration steps, the system was subjected to MD production run for 100 ns at 310 K with modified Berendsen thermostat (V-rescale) for temperature coupling and Parrinello-Rahman for pressure coupling. As a result of the MD runs, the root-mean-square deviation (RMSD) and Radius of Gyration (R_g_) of the simulated protein-ligand systems were investigated with the corrected trajectories of the systems.

## 3. Results and discussion

### 3.1. Energy minimizations of ligands

**Figure 2:**
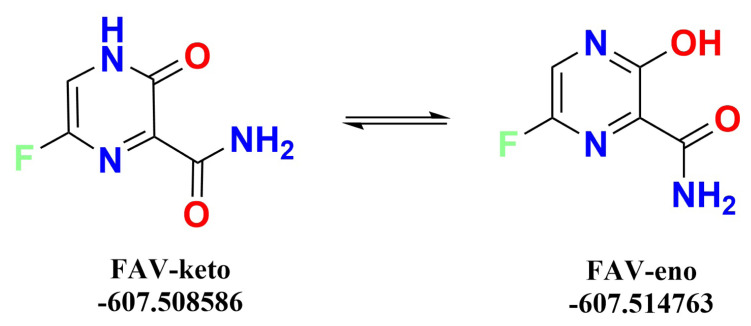
Keto enol tautomerism of favipiravir in water and corresponding energies of minimized structures.

Prior to docking studies and MD simulations, all ligands were optimized in an explicit solvent model, namely in water, to mimic the physiological environment. After successfully optimizing the ligand molecules and obtaining the lowest energy conformation of the ligands, these geometries were selectively chosen for further studies. Besides these routine minimizations, it has come to our attention that the favipiravir is capable of keto-enol tautomerism (Figure 2). For this reason, we optimized both the keto and enol forms of the molecule in water and calculated the relative energy difference between these species.

It was found that the relative energy difference between the keto and enol form favipiravir is −3.88 kcal mol^−1^. The enol form is energetically more favorable when optimized in water under the same conditions. In consequence, we chose to go further with the enol form as a result of the successful validation of the energy minimization.

### 3.2. Ligand-protein docking

All thioxanthone based molecules, in conjunction with favipiravir (keto and enol forms) and hydroxychloroquine, that were subjected to docking studies are summarized in Table 1 with the corresponding abbreviations, molecular formulas, 2D molecular structures, molecular weights, and IUPAC names.

Molecular structures of all thioxanthone based molecules bear a thioxanthone core and differentiate within each other with the attached functional groups and/or position of the functional groups. This enables us to be more flexible when modifying the thioxanthone core for desired purposes. All thioxanthone core bearing molecules in this study are the ones modified with the functional groups that are capable of hydrogen bonding and/or intermolecular interactions in a dynamic environment.

**Figure 3:**
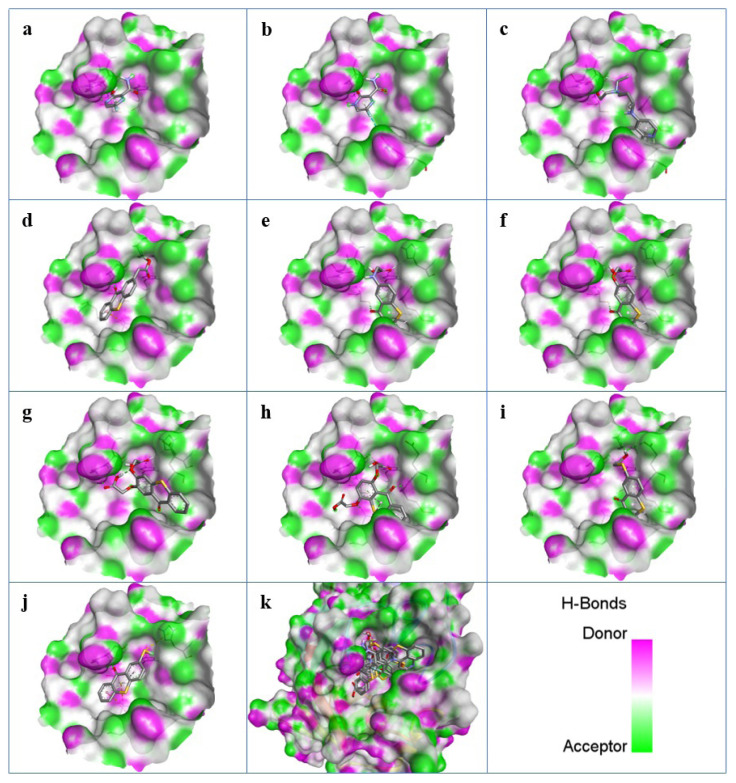
H-Bonds surfaces and 3D positions of the docked ligands: (a) FAV-eno, (b) FAV-keto, (c) HCQ, (d) TX-AA, (e) TX-NH-AA, (f) TX-O-AA, (g) *o-*TX-O-DiAA, (h) *p-*TX-O-DiAA, (i) TX-S-AA, (j) TX-SH, and (k) superimpose of all ligands.

As can be seen from Figure 3, all ligands were docked to the substrate binding site, the cavity of the M^pro^, successfully. The binding energies of all thioxanthone bearing molecules possessed a similar trend and were found to have significantly better binding energies than the known drugs favipiravir and hydroxychloroquine (Table 2).

**Figure 4:**
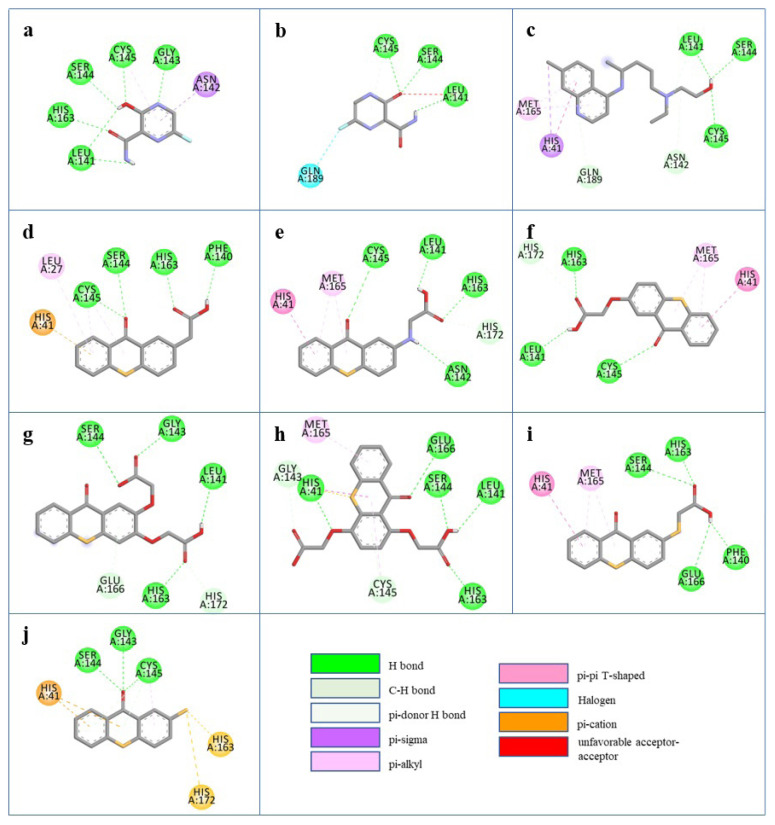
2D interactions of ligands: (a) FAV-eno, (b) FAV-keto, (c) HCQ, (d) TX-AA, (e) TX-NH-AA, (f) TX-O-AA, (g) *o-*TX-O-DiAA, (h) *p-*TX-O-DiAA, (i) TX-S-AA, (j) TX-SH with the M^pro^.

All possible intermolecular and intramolecular interactions of the ligands with the M^pro^ are given in Figure 4. It has been found that all molecules were capable making of at least 3 conventional hydrogen bonds in a combination and/or coexistence of C-H bonds, π-donor H bonds, π - σ interactions, π-alkyl interactions, π - π T-shaped interactions, interactions with a halogen and π-cation interactions.

We chose 3 thioxanthone core bearing molecules, enol form of favipiravir, and HCQ to further study with molecular dynamics simulations. TX-NH-AA was chosen due to the -NH functional group, 4 conventional hydrogen bonds in combination with a C-H bond, π-alkyl interaction, and π- π T-shaped interaction. Within the predominantly oxygen-based functional groups containing thioxanthone derivatives, *p-*TX-O-DiAA was chosen due to double acetic acid functionality, opposite positioning of the functional groups, 5 conventional hydrogen bonds in combination with a C-H bond, and a π-alkyl interaction. Among the other sulfur-containing thioxanthones, TX-SH was chosen due to the exceptional interaction characteristics, such as 3 conventional hydrogen bonds and 3 π-cation interactions.

### 3.3. ADMET and drug likeness

**Figure 5:**
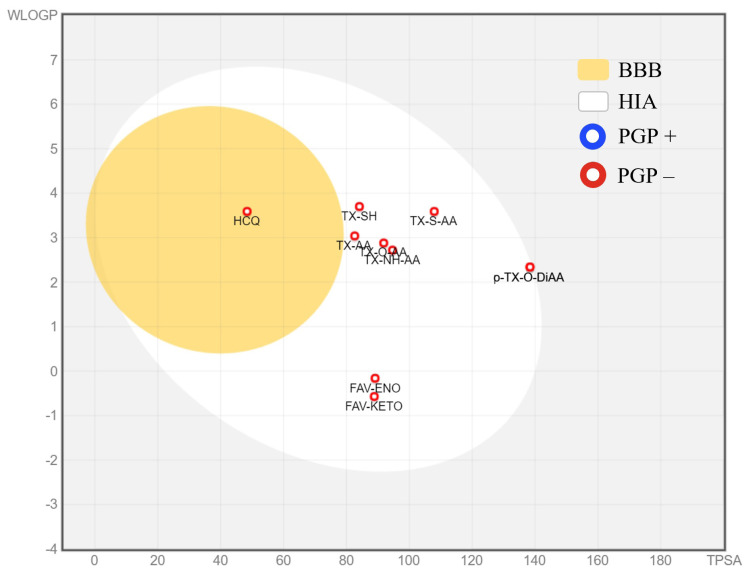
BOILED-Egg representation of the studied ligands.

In Figure 5, Brain Or IntestinaL EstimateD permeation method (BOILED-Egg) [37] representation of the studied ligands is represented. BOILED-Egg representation is a predictive model that works by computing the lipophilicity and polarity of small molecules.

Points located in the egg’s yolk are molecules predicted to passively permeate through the blood-brain barrier (BBB), whilst the ones located in the egg’s white are molecules predicted to be passively absorbed by the gastrointestinal tract, in other words, human intestinal absorption (HIA). On the other hand, blue-white circles are the representation of molecules that are predicted to be effluated from the central nervous system (CNS) by the P-glycoprotein (PGP+), and the red-white circles represent the ones that are not to be effluated from the CNS by P-glycoprotein (PGP-).[ZCN1]

As can be seen from Figure 5, all molecules except HCQ are located outside the egg’s yolk indicating that, these molecules are predicted not to passively permeate through the BBB. However, all thioxanthone based molecules and both forms of favipiravir are predicted to be passively absorbed by the gastrointestinal tract. Note that the *p-*TX-O-DiAA and *o-*TX-O-DiAA were positioned on the same spot inside the egg’s white.

BOILED-Egg representation related values of studied ligands and various molecular, lipophilicity, water solubility, pharmacokinetics, drug-likeness, medicinal chemistry, and toxicity properties of the studied molecules are provided in Table 3. As can be followed from the obtained values all studied thioxanthone based ligands showed superior properties to be utilized as drug analogues, with a sufficient number of H acceptors/donors and rotatable bonds, with good lipophilicity index values, with high gastrointestinal absorption whereas no BBB permeation, with Lipinski drug-likeness rules compatibility and good bioavailability scores, with compatible medicinal chemistry properties, good synthetic accessibility values, and good toxicity assessments.

### 3.4. Molecular dynamics (MD) simulations

**Figure 6:**
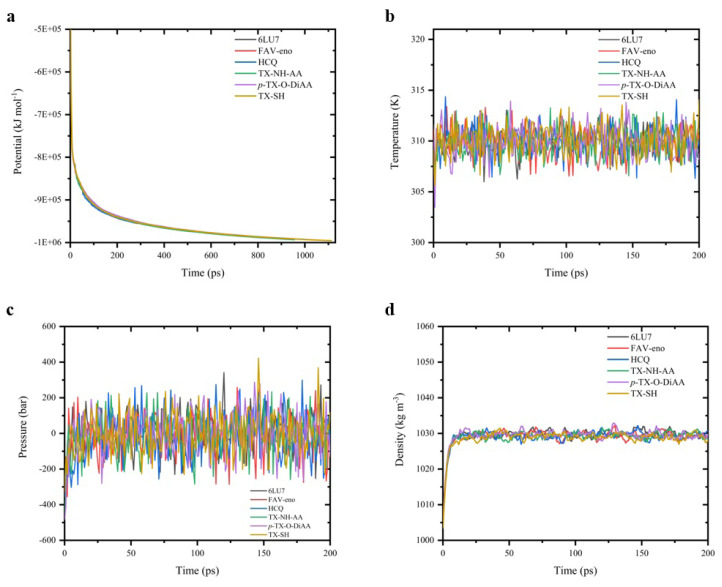
Energy minimization and equilibration properties: **a)** potential (kJ mol^−1^), **b)** temperature (K), **c)** pressure (bar), **d)** density (kg m^−3^).

To ensure that the created systems have no steric clashes or inappropriate geometry, systems were relaxed by energy minimization. After this process, it was found that the potential energy values were negative and had the order of 10^5^–10^6^ which is dependent on the size of the system and the number of ions and water molecules added to the system (Figure 6a). Additionally, the maximum force (F_max_) values were found to be smaller than 1000 kJ mol^−1^ which was targeted at the beginning energy minimization process. Thus, it was concluded that the systems have a reasonable starting structure regarding the geometry of the components and solvent orientation.

Before the actual MD production run, the system must be further optimized by equilibrating the solvent and the added ions around the protein. Otherwise, the system may produce insufficient and unreliable data. The main reason is that the solvent is generally optimized within itself and not with the solute. It needs to be brought to the temperature we would like to run the simulation and have a proper orientation around the protein. So, the first approach is to arrive at the target temperature and check the stability. Then, the pressure is applied until the proper and stable density is reached.

The first phase of the equilibration was conducted under NVT ensemble (constant number of particles, volume, and temperature), also called as “isothermal-isochoric” or “canonical” ensemble. In general, the 100 ps timeframe for this procedure is accepted to be sufficient but depending on the system properties this time can be prolonged. For the NVT ensemble, we prolonged the timeframe up to 200 ps. It can be followed in Figure 6b that the temperature of the system rapidly reached the target value (310 K) and remained stable during the equilibration for all the studied systems.

The second phase of the equilibration was conducted under NPT ensemble (constant number of particles, pressure, and temperature), also referred to as “isothermal-isobaric” ensemble. We prolonged once again the timeframe up to 200 ps. As can be seen from Figure 6c the pressure fluctuated rapidly and widely during the equilibration, as expected. However, the density (Figure 6d) reached a certain value (approx. 1030 kg m^−3^) and remained stable over the course of the equilibration for all the studied systems.

**Figure 7:**
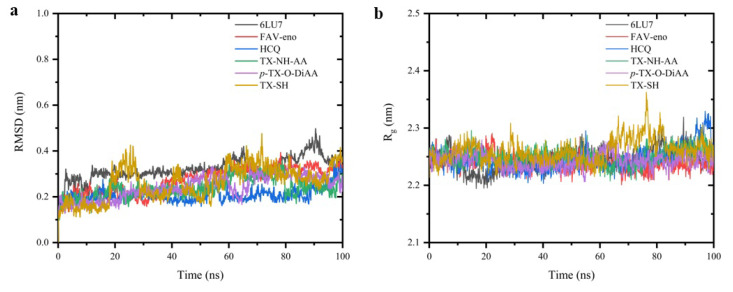
**a)** RMSD of backbone and **b)** radius of gyration of the protein, during the MD production run for the complexes studied.

After the verification of the preoptimization and energy minimization steps for all created systems, molecular dynamics simulations were produced for 100 ns. As can be seen from Figure 7a the RMSD values for all systems fluctuated in the range of 0.1 nm (1 Å) indicating that the systems remained very stable over the course of the simulations. It should be noted that the 6LU7, the neat protein structure, showed an increased fluctuation between the time frame around 85–90 ns but became stable later. Standard antiviral drugs, FAV-eno and HCQ showed similar trends with the thioxanthone derivatives. For all studied ligand-protein systems, the rapid fluctuation as the neat protein around 90 ns was not observed. However, these initial findings must also be confirmed with the manual investigation of the produced trajectories. After the examination of the produced trajectories, it was found that HCQ, TX-NH-AA, and *p-*TX-O-DiAA are stable and located in the binding pocket of M^pro^ making strong interactions with the protein during the entire simulation. On the other hand, FAV-eno loses its stability after a short time. TX-SH possesses interesting behavior as it is stable in the binding pocket for some time but eventually loses its position but repositions itself near the binding site. This observation is in good agreement with the RMSD results of TX-SH in which it showed two unstable behaviors around 20–30 ns and 60–80 ns. However, these fluctuations were limited and have also remained in the range of 0.1–0.2 nm.

The radius of gyration (R_g_) of protein can be summarized as the compactness of the protein. If a stable folding is achieved one can expect a stable R_g_ value. On the other hand, if there is an unfolding, the R_g_ value is expected to change over time. For all the studied ligand-protein systems, the R_g_ values stayed stable (Figure 7b) except for TX-SH. TX-SH showed a sharp fluctuation in the range of 60–80 ns which is in correlation with the RMSD findings of the same system and manual observations on the trajectory.

**Figure 8:**
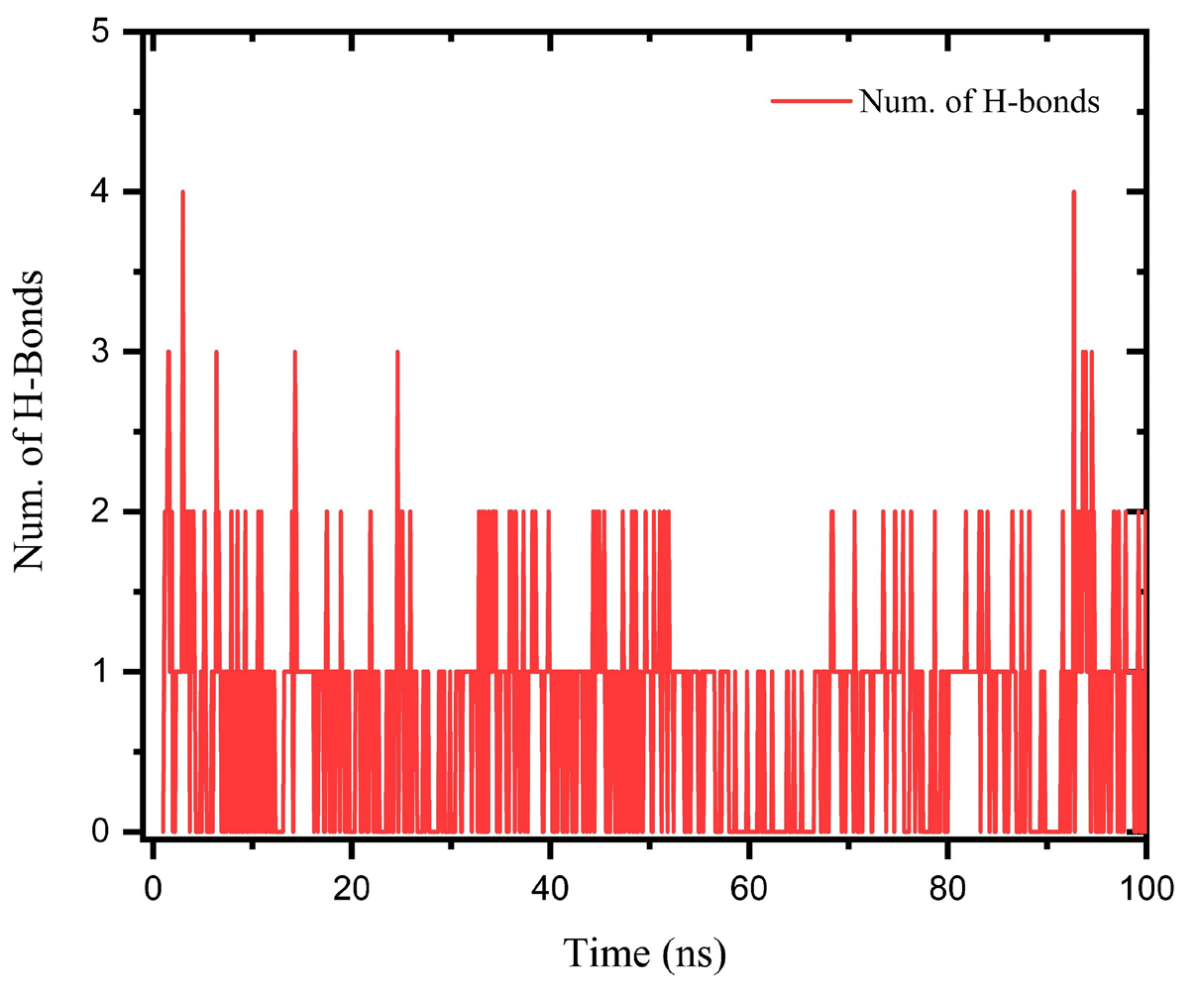
Number of H-bonds vs. time for MD run of *p*-TX-O-DiAA.

The number of H-bonds vs. time for MD run of *p*-TX-O-DiAA is given in Figure 8 suggesting a minimum of at least 1 H bond over the course of the simulation. This finding supports the other obtained results. Hence, after the investigation of all produced MD runs and consequent analysis of the trajectories, we concluded that among all studied molecules *p*-TX-O-DiAA is the most stable and has more interactions in the binding pocket of M^pro^.

## 4. Conclusion

In this study, we investigated some members of versatile thioxanthone derivatives and tried to understand the potential use of these compounds in the research against COVID-19. Almost all the studied thioxanthone based molecules have demonstrated that they can establish stable interactions with the main protease or even further stabilize the protein. Due to their high fluorescent properties as reported in the literature, we believe that these compounds have great potential to be utilized as fluorescent main protease targeting compounds. This feature, in conjunction with the stable interactions with the main protease, enables a great potential for these compounds to be used for detecting, analyzing, and preventing COVID-19. Also, it is believed that these compounds have also a great potential to be one of the key components of biomedical devices that target the main protease and as well as require a fluorescent feature. At least for the reasons mentioned above, we believe that the wide range of scientific disciplines can benefit from these initial findings with a promising opportunity for alternative modifications and further developments.

## Figures and Tables

**Table 1:** Ligand abbreviations, chemical formulas, molecular structures, molecular weights, and IUPAC names of the studied ligands.

Ligand	Chemical formula	Structure	Molecular weight (g mol^−1^)	IUPAC name
FAV-eno	C_5_H_4_FN_3_O_2_	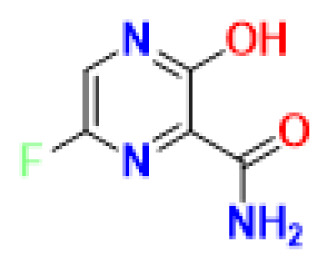	157.10	6-fluoro-3-hydroxypyrazine-2-carboxamide
FAV-keto	C_5_H_4_FN_3_O_2_	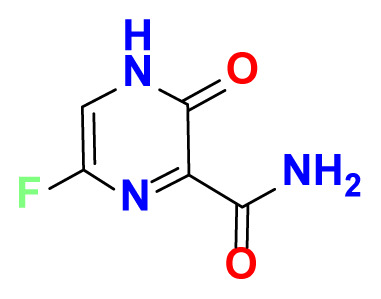	157.10	6-fluoro-3-oxo-3,4-dihydropyrazine-2-carboxamide
HCQ	C_18_H_26_ClN_3_O	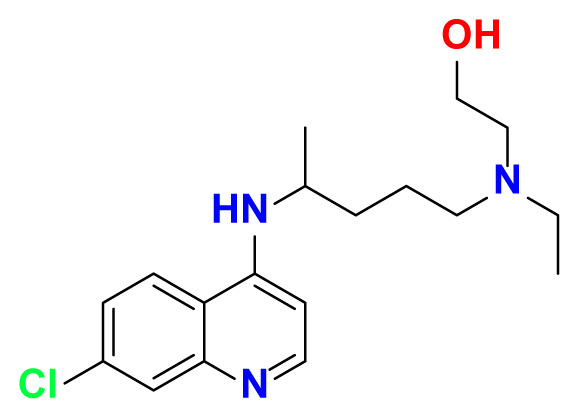	335.87	2-((4-((7-chloroquinolin-4-yl) amino) pentyl) (ethyl) amino)ethan-1-ol
TX-AA	C_15_H_10_O_3_S	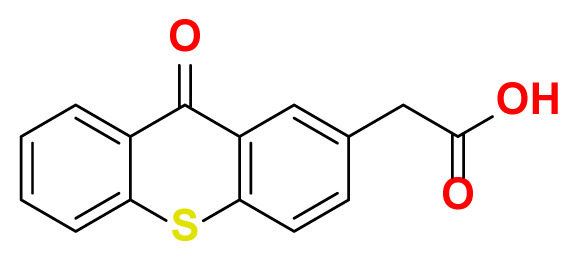	270.30	2-(9-oxo-9H-thioxanthen-2-yl) acetic acid
TX-NH-AA	C_15_H_11_NO_3_S	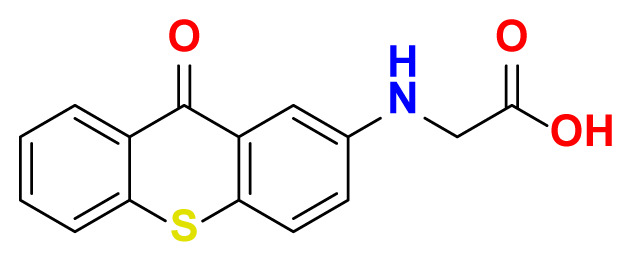	285.32	(9-oxo-9H-thioxanthen-2-yl) glycine
TX-O-AA	C_15_H_10_O_4_S	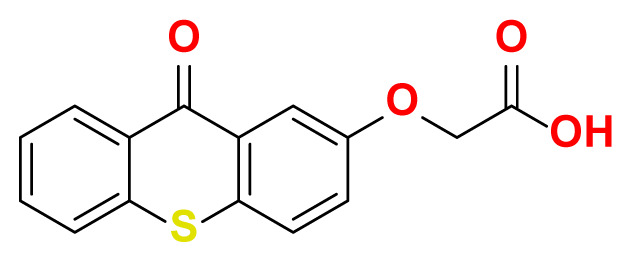	286.30	2-((9-oxo-9H-thioxanthen-2-yl) oxy) acetic acid
*o*-TX-O-DiAA	C_17_H_12_O_7_S	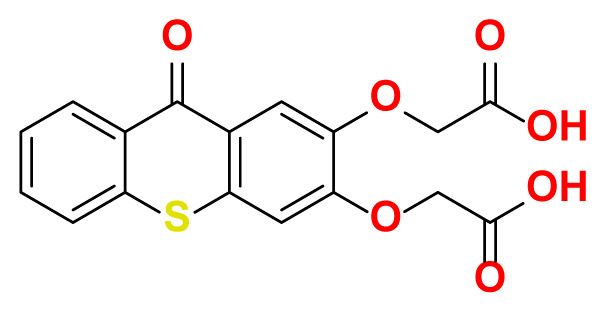	360.34	2,2′-((9-oxo-9H-thioxanthene -2,3-diyl)bis (oxy))diacetic acid
*p*-TX-O-DiAA	C_17_H_12_O_7_S	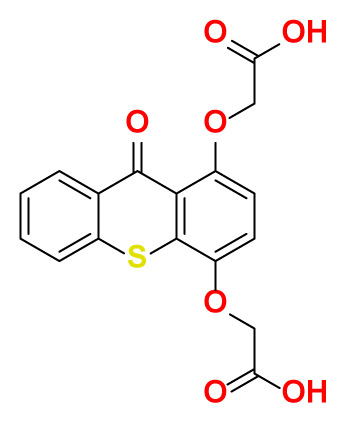	360.34	2,2′-((9-oxo-9H-thioxanthene -1,4-diyl)bis (oxy))diacetic acid
TX-S-AA	C_15_H_10_O_3_S_2_	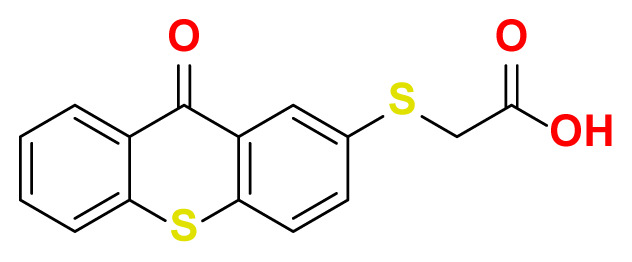	302.37	2-((9-oxo-9H-thioxanthen-2-yl) thio)acetic acid
TX-SH	C_13_H_8_OS_2_	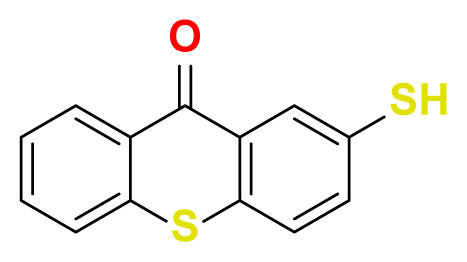	244.33	2-mercapto-9H-thioxanthen-9-one

**Table 2:** Binding free energies of the docked ligands.

Ligand	Binding energy (kcal mol^−1^)	Standard deviation	Heavy atom count	Ligand efficiency (Ligand efficiency = Docking score/Heavy atom count)
FAV-eno	−5.3	0.0471	11	−0.4818
FAV-keto	−4.9	0.0433	11	−0.4454
HCQ	−6.4	0.0000	23	−0.2782
TX-AA	−7.4	0.1247	19	−0.3894
TX-NH-AA	−7.4	0.2828	20	−0.37
TX-O-AA	−7.2	0.2357	20	−0.36
*o*-TX-O-DiAA	−7.1	0.0000	25	−0.284
*p*-TX-O-DiAA	−7.5	0.0000	25	−0.3
TX-S-AA	−7.1	0.0707	20	−0.355
TX-SH	−6.4	0.0471	16	−0.4

**Table 3:** Molecular, lipophilicity, water solubility, pharmacokinetics, drug likeness, medicinal chemistry, and toxicity properties of the studied molecules.

Ligand	Smiles codes	#H acceptor	#H donor	#Rotatable bonds	Lipophilicity					
					iLogP	XLogP3	WLogP	MlogP	Silicos-IT	Consensus Log P_o/w_
**FAV-eno**	FC1=CN=C(O)C(C(N)=O)=N1	5	2	1	0.70	−0.04	−0.16	−1.36	0.06	−0.16
**FAV-keto**	FC(N=C1C(N)=O)=CNC1=O	4	2	1	0.39	−0.56	−0.57	−1.30	0.69	−0.27
**HCQ**	CC(CCCN(CCO)CC) NC1=CC=NC2=CC(Cl)=CC=C21	3	2	9	3.58	3.58	3.59	2.35	3.73	3.37
**TX-AA**	O=C1C2=C(C=CC(CC(O)=O)=C2) SC3=CC=CC=C31	3	1	2	1.93	3.45	3.04	2.44	4.46	3.06
**TX-NH-AA**	O=C1C2=C(C=CC(NCC(O)=O)=C2) SC3=CC=CC=C31	3	2	3	1.83	3.57	2.72	−0.36	3.67	2.29
**TX-O-AA**	O=C1C2=C(C=CC(OCC(O)=O)=C2) SC3=CC=CC=C31	4	1	3	2.13	3.56	2.88	1.86	3.99	2.88
***o*** **-TX-O-DiAA**	O=C1C2=C(C=C(OCC(O)=O) C(OCC(O)=O)=C2)SC3=CC=CC=C31	7	2	6	1.57	2.63	2.34	0.91	3.36	2.16
***p*** **-TX-O-DiAA**	O=C1C2=C(C(OCC(O)=O)=CC=C2OCC(O)=O) SC3=CC=CC=C31	7	2	6	1.55	2.63	2.34	0.91	3.36	2.16
**TX-S-AA**	O=C1C2=C(C=CC(SCC(O)=O)=C2) SC3=CC=CC=C31	3	1	3	2.09	4.10	3.59	2.71	4.55	3.41
**TX-SH**	O=C1C2=C(C=CC(S)=C2)SC3=CC=CC=C31	1	0	0	2.41	4.14	3.70	3.26	4.99	3.70

Ligand	Water Solubility						Pharmacokinetics						
	LogS (ESOL)	Class	LogS (Ali)	Class	LogS (Silicos-IT)	Class	GI absorption	BBB permeant	P-gp substrate	CYP1A2 inhibitor	CYP2C19 inhibitor	CYP2C9 inhibitor	CYP2D6 inhibitor
**FAV-eno**	−1.13	Very Soluble	−1.38	Very Soluble	−0.93	Soluble	High	No	No	No	No	No	No
**FAV-keto**	−0.80	Very Soluble	−0.84	Very Soluble	−1.42	Soluble	High	No	No	No	No	No	No
**HCQ**	−3.91	Soluble	−4.28	Moderately Soluble	−6.35	Poorly Soluble	High	Yes		No Yes	No	No	Yes
**TX-AA**	−4.10	Moderately Soluble	−4.87	Moderately Soluble	−5.12	Moderately Soluble	High	No	No	Yes	No	Yes	No
**TX-NH-AA**	−4.18	Moderately Soluble	−5.24	Moderately Soluble	−5.17	Moderately Soluble	High	No	No	Yes	No	Yes	No
**TX-O-AA**	−4.18	Moderately Soluble	−5.17	Moderately Soluble	−4.86	Moderately Soluble	High	No	No	Yes	No	Yes	No
***o*** **-TX-O-DiAA**	−3.75	Soluble	−5.19	Moderately Soluble	−4.33	Moderately Soluble	High	No	No	No	No	Yes	No
***p*** **-TX-O-DiAA**	−3.75	Soluble	−5.19	Moderately Soluble	−4.33	Moderately Soluble	High	No	No	No	No	Yes	No
**TX-S-AA**	−4.62	Moderately Soluble	−6.07	Poorly Soluble	−5.22	Moderately Soluble	High	No	No	Yes	Yes	Yes	No
**TX-SH**	−4.61	Moderately Soluble	−5.61	Moderately Soluble	−5.46	Moderately Soluble	High	No	No	Yes	Yes	Yes	No

Ligand	Pharmacokinetics		Druglikeness						Medicinal Chemistry			
	CYP3A4 Inhibitor	LogK_P_ (Skin permeation) (cm s^−1^)	Lipinski	Ghose	Veber	Egan	Muegge	Bioavailability score	PAINS	Brenk	Leadlikeness	Synthetic accessibility
**FAV-eno**	No	−7.29	Yes	No	Yes	Yes	No	0.55	0	0	No	2.03
**FAV-keto**	No	−7.66	Yes	No	Yes	Yes	No	0.55	0	0	No	2.08
**HCQ**	No	−5.81	Yes	Yes	Yes	Yes	Yes	0.55	0	0	No	2.82
**TX-AA**	No	−5.50	Yes	Yes	Yes	Yes	Yes	0.56	0	1	Yes	2.26
**TX-NH-AA**	No	−5.51	Yes	Yes	Yes	Yes	Yes	0.56	0	1	No	2.33
**TX-O-AA**	No	−5.52	Yes	Yes	Yes	Yes	Yes	0.56	0	1	No	2.45
***o*** **-TX-O-DiAA**	No	−6.63	Yes	Yes	Yes	No	Yes	0.56	0	1	No	2.94
***p*** **-TX-O-DiAA**	No	−6.63	Yes	Yes	Yes	No	Yes	0.56	0	1	No	3.28
**TX-S-AA**	No	−5.23	Yes	Yes	Yes	Yes	Yes	0.56	0	1	No	2.71
**TX-SH**	No	−4.85	Yes	Yes	Yes	Yes	Yes	0.55	0	2	No	2.30

Ligand	Toxicity									
	Ames Toxicity	Max. Tolerated Dose (human) (Log mg kg^−1^ day^−1^)	hERG I Inhibitor	hERG II Inhibitor	Oral Rat Acute Toxicity (LD50) (mol kg^−1^)	Oral Rat Chronic Toxicity (LOAEL) (Log mg kg_bw^−1^ day^−1^)	Hepatotoxicity	Skin sensitisation	*T.Pyriformis* Toxicity (Log μg L^−1^)	Minnow Toxicity (Log mM)
**FAV-eno**	No	1.163	No	No	1.658	1.718	No	No	−0.118	3.447
**FAV-keto**	No	1.116	No	No	1.916	1.254	No	No	−0.433	3.63
**HCQ**	No	0.621	No	Yes	2.589	1.106	Yes	No	0.779	2.751
**TX-AA**	No	0.847	No	No	2.249	1.54	Yes	No	0.301	−1.025
**TX-NH-AA**	No	0.577	No	No	2.292	1.463	No	No	0.285	−0.528
**TX-O-AA**	Yes	0.413	No	No	2.289	0.971	No	No	0.298	−0.535
***o*** **-TX-O-DiAA**	No	−0.094	No	No	2.491	2.132	Yes	No	0.285	−0.153
***p*** **-TX-O-DiAA**	No	−0.068	No	No	2.516	1.811	No	No	0.285	0.619
**TX-S-AA**	No	0.715	No	No	2.262	1.977	Yes	No	0.321	−1.322
**TX-SH**	Yes	0.557	No	Yes	1.814	1.289	Yes	Yes	1.281	−0.798

## Footnotes

1 WHO (2022). WHO Coronavirus (COVID-19) Dashboard [online]. Website https://covid19.who.int/ [accessed 01 April 2022].
